# Ethanol Neurotoxicity in the Developing Cerebellum: Underlying Mechanisms and Implications

**DOI:** 10.3390/brainsci3020941

**Published:** 2013-06-14

**Authors:** Ambrish Kumar, Holly A. LaVoie, Donald J. DiPette, Ugra S. Singh

**Affiliations:** 1 Department of Pathology, Microbiology and Immunology, School of Medicine, University of South Carolina, Columbia, SC 29209, USA; E-Mail: ambrish.kumar@uscmed.sc.edu; 2 Department of Cell Biology and Anatomy, School of Medicine, University of South Carolina, Columbia, SC 29209, USA; E-Mail: holly.lavoie@uscmed.sc.edu; 3 Department of Internal Medicine, School of Medicine, University of South Carolina, Columbia, SC 29209, USA; E-Mail: donald.dipette@uscmed.sc.edu

**Keywords:** alcohol neurotoxicity, synaptogenesis, cerebellum, fetal alcohol spectrum disorders, oxidative stress

## Abstract

Ethanol is the main constituent of alcoholic beverages that exerts toxicity to neuronal development. Ethanol affects synaptogenesis and prevents proper brain development. In humans, synaptogenesis takes place during the third trimester of pregnancy, and in rodents this period corresponds to the initial few weeks of postnatal development. In this period neuronal maturation and differentiation begin and neuronal cells start migrating to their ultimate destinations. Although the neuronal development of all areas of the brain is affected, the cerebellum and cerebellar neurons are more susceptible to the damaging effects of ethanol. Ethanol’s harmful effects include neuronal cell death, impaired differentiation, reduction of neuronal numbers, and weakening of neuronal plasticity. Neuronal development requires many hormones and growth factors such as retinoic acid, nerve growth factors, and cytokines. These factors regulate development and differentiation of neurons by acting through various receptors and their signaling pathways. Ethanol exposure during development impairs neuronal signaling mechanisms mediated by the *N*-methyl-d-aspartate (NMDA) receptors, the retinoic acid receptors, and by growth factors such as brain-derived neurotrophic factor (BDNF), insulin-like growth factor 1 (IGF-I), and basic fibroblast growth factor (bFGF). In combination, these ethanol effects disrupt cellular homeostasis, reduce the survival and migration of neurons, and lead to various developmental defects in the brain. Here we review the signaling mechanisms that are required for proper neuronal development, and how these processes are impaired by ethanol resulting in harmful consequences to brain development.

## 1. Introduction

**Figure 1:**
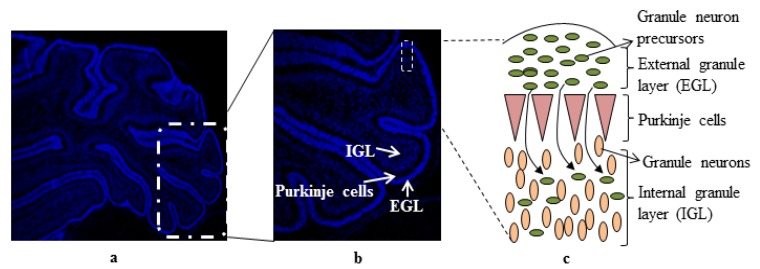
Cross section of postnatal day seven rat cerebellum (**a**), showing external granule layer (EGL), Purkinje cells and internal granule layer (IGL) (**b**). During cerebellar development, granule cell precursors (GCPs) present in EGL proliferate and differentiated into mature granule neurons. Mature neurons start migrating past the Purkinje cell layer to form the internal granule layer (IGL) (**c**).

Prenatal ethanol exposure interferes with the synaptogenesis phase of brain development, especially within the cerebellum and leads to various impairments in brain function [1,2]. In humans, synaptogenesis begins during the third trimester of pregnancy and continues through the first few years of life. In rodents, this period corresponds to postnatal days four to nine (P4–P9). Although neuronal development in all areas of brain is affected, the cerebellum is more susceptible to the harmful effects of ethanol. A single exposure of ethanol to rat pups depletes Purkinje cells on postnatal days four to six and cerebellar granule neurons on postnatal days six to eight [3,4,5,6,7,8]. It has been suggested that the endogenous levels of antioxidants in the cerebellum and hippocampus, being lower when compared to other areas of the brain, makes them more susceptible to ethanol’s teratogenic effects [9,10]. During the course of cerebellar development, Purkinje cells and cerebellar granule neuron (CGNs) are developmentally regulated [11,12]. The CGNs are generated on the outer surface of cerebellum and form the external granule layer (EGL). Granule neuron precursors (GNPs) which give rise to CGNs first extensively proliferate; some GNPs exit the cell cycle and then start differentiating into mature CGNs. On postnatal days six to eight in rats, GNPs start migrating past the Purkinje cell layer to form the internal granule layer (IGL) (Figure 1). The migration, differentiation, and maturation of CGNs are regulated by various factors which include genes involved in cell cycle regulation, receptors (e.g., the *N*-methyl-d-aspartate (NMDA) receptors and the retinoic acid receptors), and nerve growth factors such as brain-derived neurotrophic factor (BDNF), insulin-like growth factor 1 (IGF-I), and basic fibroblast growth factor (bFGF) [13,14,15,16,17]. Ethanol exposure impairs these receptors and associated signaling pathways and, as a result, CGNs fail to migrate out of the external granule layer and undergo apoptoic cell death (also reviewed previously by Luo [18]).

Purkinje cells originate on embryonic days 13–16 of rat brain development. Before and after birth, these cells are dispersed over the surface of the cerebellar cortex. On postnatal day five, they start aligning as a monolayer and synaptogenesis occurs into the third and fourth weeks postnatally. Purkinje cell loss in response to ethanol exposure is extensive in the early developing lobules (I–III, and VIII–X) with little or no loss in the later developing lobules (IV–VII) [19,20]. Ethanol also induces neuronal loss in other areas of the brain. Bonthius and West reported ethanol-induced neuronal loss in the hippocampus in addition to the cerebellum [7]. In their experiments Sprague-Dawley rat pups were exposed to ethanol between postnatal days 4–10. At this time point ethanol significantly reduced the neuronal number in the CA1 region of the hippocampus but not in CA3, CA4, and dentate gyrus. The cytochrome C release from mitochondria, activation of caspase-3, degradation of DNA, and induction of programmed cell death (apoptosis) are the main events that are induced by ethanol to prevent neuronal development in different areas of brain [21]. 

Various mechanisms including generation of oxidative stress, disruption of protein synthesis, growth factors and cytokines, and interaction with membrane and nuclear receptors have been reported to bring about the neurotoxic effects of ethanol. In this review, we summarize the mechanisms/pathways and their downstream targets, particularly in cerebellum, that may be responsible for inducing neurotoxic effects of ethanol.

## 2. Ethanol Increases Oxidative Stress and Induces Apoptotic Cell Death

**Figure 2:**
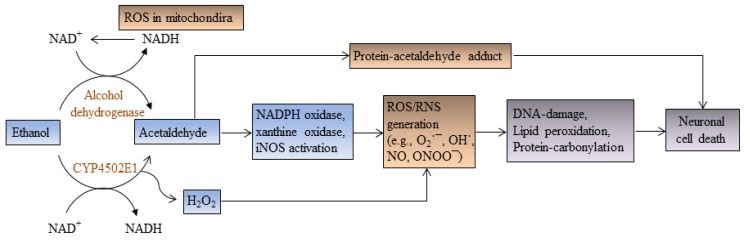
In ethanol metabolism, the enzyme alcohol dehydrogenase oxidizes ethanol to acetaldehyde, while cytochrome P450-2E1 enzyme converts ethanol to acetaldehyde and H_2_O_2_. Acetaldehyde interacts with proteins and forms protein-acetaldehyde adducts (acetaldehyde-hemocyanin adduct). Hydrogen peroxide and acetaldehyde (via transcriptional activation of NADPH oxidase, xanthine oxidase, and iNOS) generate free radicals (reactive oxygen species, ROS/reactive nitrogen species, RNS), which oxidize proteins, lipids, and DNA leading to apoptotic cell death in the developing cerebellum.

Under normal physiological conditions, a proper balance between free radicals (reactive oxygen species, ROS, and reactive nitrogen species, RNS) and the levels of antioxidants is required for cell survival. Increased generation of ROS/RNS (such as superoxide anion, hydroxyl radical, and peroxynitrite, *etc.*), and failure of antioxidative mechanisms (including superoxide dismutase, catalase, glutathione peroxidase, *etc.*) to remove excess ROS/RNS generates oxidative stress. Increased levels of free radicals damage DNA, oxidize cellular proteins and lipids, and disrupt the membrane permeability of mitochondria. Oxidative damage of mitochondria releases cytochrome C and activates caspase pathways, which lead to cell death. Various *in vitro*, as well as *in vivo*, data suggest that prenatal and postnatal ethanol induces elevated level of oxidative stress either by generation of free radicals (ROS/RNS) or disruption of antioxidative defense mechanisms and, thereby, promotes apoptotic cell death in the cerebellum of rodent brains [5,22,23,24,25,26,27,28,29]. Ethanol exposure to *in vitro* cultures of cortical neurons [26] and fetal rhombencephalic neurons [30] generates ROS and induces mitochondrial membrane depolarization and apoptosis (Figure 2). Pretreatment of cultured fetal cortical neurons with *N*-acetylcysteine inhibits ethanol-mediated reduction in cellular glutathione level and prevents cell death, indicating a role for oxidative stress in ethanol toxicity [26].

Alcohol dehydrogenase (ADH) oxidizes ethanol to acetaldehyde, while cytochrome p450-2E1 (CYP2E1) converts ethanol to acetaldehyde and H_2_O_2_. The presence of CYP2E1 has been reported in neurons within the cerebral cortex, Purkinje and granule cell layers of the cerebellum, pyramidal neurons in hippocampal CA1, CA2, and CA3 regions in rat and human brains. Ethanol exposure to primary cortical neurons, isolated from human fetal brain tissue, moderately increased the enzymatic activity of ADH, whereas the expression and activity of CYP2E1 increased significantly. The increased levels of ethanol metabolites (acetaldehyde and H_2_O_2_) generate ROS and NO via the activation of NADPH/xanthine oxidase and inducible nitric oxide synthase (iNOS) enzyme pathways. Addition of apocyanin, an NADPH inhibitor, and allopurinol, a xanthine oxidase inhibitor, inhibits free radical production in ethanol exposed neuronal cultures, suggesting the direct involvement of NADPH/xanthine oxidase pathways in ROS/RNS generation by ethanol in neurons [31]. 

In addition, ethanol also increases ROS levels by reducing antioxidative enzyme levels via disrupting the nrf2-mediated pathways [22]. NF-E2 related factor-2 (nrf2) protein, a transcription factor, promotes the transcription of cytoprotective genes such as catalase, superoxide dismutase (SOD), UDP-glucuronosyltransferase, NADPH quinine oxidoreductase 1 (NQO1), heme oxygenase 1, glutathione peroxidase, and γ-glutamylcysteine synthetase that protect cells from oxidative damage. Under normal conditions, nrf2 remains associated with the protein keap1 in the cytoplasm. In the presence of oxidative insults, the nrf2/keap1 complex dissociates and nrf2 translocates to the nucleus where it interacts with antioxidant response elements (AREs) present upstream of nrf2-regulated genes and increases expression of these detoxifying genes, which then remove excess ROS and protect the cells from oxidative damage [32]. Our laboratory demonstrated that ethanol exposure to postnatal day seven rat pups reduces the nuclear abundance and DNA-binding activity of nrf2 to an ARE and down-regulates the expression of nrf2-regulated genes products, SOD and NQO1, in the cerebellum. Lower levels of antioxidant enzymes and glutathione resulting from ethanol exposure thus fail to remove the ROS in neurons [22]. The resulting oxidative stress oxidizes lipids (as measured by increased level of 4-HNE) and DNA (as measured by increased levels of 8-OHdG). These effects disturb mitochondrial membrane potential and lead to the activation of caspase-3 and cell death. Heaton *et al.* [33] have suggested that ethanol-activated JNK (by phosphorylation) dissociates Bax:14-3-3 protein complex in the cytosol, and that released Bax protein translocates to the mitochondrial membrane in postnatal day four rat CGNs. The interaction of Bax with the voltage-dependent anion channel (VDAC) and adenine nucleotide translocator (ANT) on mitochondrial membranes induces loss of mitochondrial integrity, releases cytochrome *c* into the cytosol, and initiates apoptotic cell death [34].

## 3. Ethanol Promotes Retinoic Acid Teratogenicity

Retinoic acid (RA), an active metabolite of vitamin A, plays an important role in the development of embryos and their central nervous system, and impairment in retinoic acid metabolism leads to various neurological disorders [35,36,37]. During retinoic acid metabolism, retinol released by the liver circulates in plasma in a bound form with retinol binding protein (RBP); RBP then releases retinol within tissues for cellular uptake. Conversion of retinol to retinoic acid is a two steps process, which involves oxidation of retinol to retinal catalyzed by the enzyme retinol dehydrogenase and subsequent retinal oxidization to retinoic acid by the enzyme retinal dehydrogenase. Retinoic acid binds to intracellular binding proteins then translocates to the nucleus. In the nucleus, RA acts as a ligand for its receptors, retinoic acid receptor (RAR), and retinoid X receptor (RXR) [38]. Each receptor has three different isoforms namely, RARα, RARβ, RARγ, and RXRα, RXRβ, RXRγ. RAR exists as a homodimer while RXRs also interact with RARs and other nuclear receptors such as the vitamin D receptor. After binding to retinoic acid, these retinoic acid receptors interact with retinoic acid response elements present in the promoter regions of their target genes, and in turn control the gene expression level for proteins such as tissue transglutaminase (TG2), D2 dopamine receptor, NMDA receptor, protein kinase C substrate neurogranin, calmodulin kinase II, and cholinergic-specific proteins.

Retinoic acid also acts as a teratogen. The altered level of RA leads to craniofacial malformation and CNS abnormalities, which are similar to ethanol effects suggesting that ethanol might interfere with the RA metabolism. Interestingly, enzymes such as alcohol dehydrogenase and cytochrome P450 are involved in both alcohol and retinoid metabolism [39]. Ethanol as a competitive inhibitor decreases RA synthesis by inhibiting enzyme retinol dehydrogenases with the exception of short-chain microsomal retinol dehydrogenase. Short-chain microsomal retinol dehydrogenase (e.g., RoDHII) has been reported in developing cerebellum. The increased level of enzymes involved in retinoic acid metabolism such as microsomal retinol dehydrogenase, the cytosolic retinal dehydrogenase, and cytochrome P450-1A1 and 1A2 by ethanol confirms the possible interference of ethanol with RA biosynthesis in mice brains [39,40,41,42,43,44]. 

Previous *in vivo* reports have shown that alcohol consumption affects RA concentration in different organs, including the brain, by increasing substrate concentration, altering retinoid-binding protein expression, and/or disrupting RA receptors [45]. Ethanol induces an increase in RA synthesis in the cerebellum, whereas RA synthesis is decreased in the cerebellar cortex of adult rats [46,47]. McCaffery *et al.* [47] reported that astrocytes are the major site for RA synthesis in postnatal developing cerebellum, and ethanol exposure increases the level of RA via ethanol-activated short-chain retinol dehydrogenase in postnatal rat cerebellum. Among the various areas of the CNS, the cerebellum is most sensitive to the effects of RA and excess RA promotes loss of granule cells in newborn rats. Besides RA metabolism, ethanol also affects the expression as well as activity of retinoic acid receptors. Our laboratory reported that ethanol administration to seven-day-old rat pups reduced the expression of RARα/γ, while it increased the expression of RXRα/γ in the cerebellum. Ethanol also significantly inhibited the DNA-binding activity of RAR and increased the activity of RXR to a consensus DNA-response element [48]. Studies have demonstrated that RXR targets genes predominantly involved in apoptosis [49,50], while RAR regulates genes promoting neuronal differentiation [51,52]. Our studies have demonstrated that exposure of CGNs to an acute ethanol dose (80 mM) *in vivo* induces apoptosis, while exposure to a moderate ethanol dose (40 mM) inhibits neurite outgrowth [53] indicating that ethanol may target RA signaling to promote harmful effects. 

## 4. Ethanol Impairs Brain-Derived Nerve Growth Factor (BDNF) Signaling

Brain-derived nerve growth factor (BDNF) plays a crucial role in synaptic plasticity, survival, growth, and maturation of neurons in early brain development as well as in adult brains [54,55]. BDNF promotes neuronal survival by the increased expression of pro-survival protein, Bcl-2 and via activation of CREB signaling pathway. Binding of BDNF to its receptor, TrkB, activates the MAP kinase, phospholipase C-γ (PLC-γ) and PI3-kinase pathways, and regulates transcriptional activity of genes involved in pro-apototic and anti-apoptotic pathways. The expression of BDNF and its receptor TrkB in the cerebellum and cerebellar granule neurons are developmentally regulated, and BDNF/TrkB interaction promotes survival, differentiation, and migration of cerebellar neurons [15,17,56,57].

Ethanol exposure modulates the expression of BDNF mRNA in different brain areas, based on the dose, administration route, and duration of ethanol exposure. Raivio *et al.* [58] reported that ethanol administration (1.2 g/kg b.wt., I.P.) to Wistar rats decreases BDNF mRNA levels in the hippocampus, while ethanol at dose of 2.5 g/kg b.wt. (I.P.) decreases BDNF mRNA levels in the frontal cortex, nucleus accumbens, and amygdala, and increases it in the ventral tegmental area. Other studies found that ethanol exposure increases BDNF mRNA levels in the dorsal striatum [59,60] and in the hippocampus [60,61] but not in frontal cortex [59,60]. Kulkarny *et al.* [61] observed that a single acute ethanol exposure (174 mg/dL) via vapor inhalation to Sprague-Dawley rats (postnatal day 23) increases the mRNA and protein level of BDNF in CA3, dentate gyrus, and the hilar region of the hippocampus, but not in the cerebellum. The observed unchanged level of cerebellar BDNF in response to ethanol may be due the age of mice at the time of ethanol exposure because the cerebellum is more sensitive to ethanol at postnatal days four to nine. Similarly, vapor inhalation of ethanol during postnatal days 10–15, increases the hippocampal BDNF mRNA level in P16 and P20 rats and decreases it in P60 rats [62]. This window of ethanol exposure (postnatal days 10–15) reduces the total number of pyramidal and hilar neurons in the hippocampus [63,64,65]. In addition, early postnatal ethanol exposure (postnatal day 4–10) elevated BDNF protein level on postnatal day 10, and returned it to control levels on postnatal day 21 in the hippocampus and cortex/striatum [66]. 

Binding of BDNF ligand to its TrkB receptor activates signaling pathways. The reduced mRNA level of both BDNF and its receptor TrkB in cerebellar Purkinje cells was observed when ethanol was administered on postnatal day four (P4) rats, but not on postnatal day nine [67,68]. Thus, ethanol exposure on P4 disrupts BDNF-TrkB neurotrophic signals that result in the loss of apoptotic suppression and thereby induces death in Purkinje cells. The BDNF-TrkB signaling cascade involves activation of ERK1/2 and PI3-kinase pathways. Ethanol exposure to offspring during the gestational and lactation period down-regulates these intracellular survival pathways, and induces apoptosis in developing cerebral cortex [69]. Another study carried out by Li *et al.* [70] in a transgenic mouse model expressing an activator protein-1 (AP-1) reporter transgene found that BDNF stimulated AP-1 activation through the PI3K/Akt/JNK pathways in CGNs isolated from five to six-day-old pups. Addition of ethanol blocks BDNF-mediated activation of PI3K/Akt/JNK pathway and AP-1 activation without affecting activation/phosphorylation of ERK1/2 and p38 [70]. Ethanol-exposure also inhibited BDNF-induced Rac1/Cdc42 activation and promotes RhoA activation in axonal cones in rat hippocampal pyramidal neurons [71] and prevents axonal growth and its guidance in the early stages of hippocampal development.

The L1 neural cell adhesion molecule, netrin1, and GDNF promote axon outgrowth by stimulating both Src family kinase (SFK) and MAP kinases/ERK1 and 2, whereas BDNF induces axon outgrowth only by activating ERK1/2. In CGNs, ethanol inhibits axon outgrowth by inhibiting sequential activation of SFK, Crk-associated substrate (Cas) and ERK1/2 [72] without affecting BDNF/ERK1/2 activation pathways suggesting that SFK is the primary target of ethanol [73].

## 5. Ethanol Impairs Cytokine Signaling

Chronic ethanol treatment stimulates glial cells, and up-regulates cytokines and inflammatory mediators in both brain and astroglial cells, activating signaling pathways and transcription factors associated with inflammation and cell death [74]. Cytokine production is initiated by signaling through toll-like receptor 4/type I interleukin-1 receptor (TLR4/IL-1RI) in response to their ligands, lipopolysaccharide (LPS) and IL-1β, and plays vital roles in inflammation, injury, and stress. Under stress and disease conditions, astrocytes are activated by IL-1β produced by glial cells. Interaction of IL-1β with its receptor, IL-1R1, stimulates downstream signaling pathways including IRAK/MAPK/NF-κB and AP-1. Activation of NF-κB and AP-1 transcription factors leads to the induction of gene encoding inducible nitric oxide synthase (iNOS) and cyclooxygenase-2 (COX-2) [75,76]. Ethanol suppresses both cytokine-induced iNOS expression in C6 rat glioma cells [77,78] and the LPS-induced increase of nitric oxide production in rat mixed glial culture [79]. Previous studies have shown that ethanol inhibits LPS-induced IL-1β expression and NF-κB activation in microglial cells [80]. Acute ethanol exposure affects both MAPK activation and cytokine production by impairing the TLR-mediated macrophage inflammatory response [81,82].

Studies carried out both in primary cultures of astrocytes prepared from rat cortex and in cerebral cortex from five-month, ethanol-fed rats show elevated levels of iNOS, COX-2, and IL-1β levels, mediators of TLR4 and IL1R1 inflammatory signaling pathways. Up-regulation of these inflammatory mediators stimulates rapid phosphorylation of IL-1R1 associated kinase (IRAK) and MAP kinases, including ERK1/2, SAPK/JNK, and p-38 pathways, which in turn trigger the activation of transcription factors NF-κB and AP-1; these effects result in activation of caspase-3 and induce apoptosis in cortex of ethanol-fed rats and in astrocytes exposed to ethanol [74,83]. Blocking of TLR4 and IL-1RI with neutralizing antibodies abolishes most of the effects of ethanol on the inflammatory signaling events and prevents cell death, suggesting that ethanol-induced inflammatory processes are mediated by the activation of TLR4/IL-1RI in brains and astrocytes. In a recent study carried out in cerebellar granule neurons isolated from five to seven-day-old mice suggests that ethanol increases intracellular Ca^2+^ and promotes cell death. Incubation of CGNs with either BAPTA/AM (a calcium chelator) or with 2-APB (an inhibitor of inositol-triphosphate receptor) blocks increased Ca^2+^ levels and cell death [84]. These studies indicated that Ca^+2^-mediated activation of Ca^2+^/calmodulin-dependent protein kinase II signaling may be the possible mechanism of action of ethanol neurotoxicity [85]. 

## 6. Ethanol Impairs IGF-I Signaling

In the cerebellum, the IGF-I receptor is expressed in both Purkinje cells and CGNs, and IGF-I, secreted from Purkinje cells, promotes survival and differentiation of CGNs [86]. Although, IGF-I mRNA expression is not observed in any stage of CGN development, Purkinje cells have peak expressions of IGF-I in postnatal days 4–10 (brain-growth spurt period) and reduced expression at postnatal day 28 in rats [14,87]. Previous studies using *in vitro* and *in vivo* models show that ethanol inhibits the insulin-mediated survival pathway and activates mitochondrial function in cerebellar neuron cells [88,89,90,91]. Chronic *in utero* exposure to ethanol throughout pregnancy inhibits insulin, IGF-I, IGF-II receptor binding, IGF-I receptor tyrosine kinase phosphorylation/activity, and increases the level of oxidative stress in cerebellum of postnatal day two pups (P2). These effects block insulin-stimulated neuronal viability, neural migration and mitochondrial function, astrocyte proliferation, and microglial activation in the cerebellum.

CGNs prepared from these ethanol-exposed pups show increased levels of oxidative stress and elevated levels of proteins p53, Fas-receptor, and Fas-ligand involved in apoptotic cell death. Ethanol also inhibits insulin-stimulated phosphorylation of Akt, GSK3β, and BAD proteins, activates the enzymatic activity of GSK3β and BAD, and reduces the level of insulin responsive genes such as glyceraldehyde 3-phosphate dehydrogenase (GAPDH) and glucose uptake transporters (GLUTs) [88,91]. Ethanol also inhibits IGF-I induced phosphorylation of IRS-1 and PI3-kinase activity in CGNs isolated from postnatal day seven rat pups [89]. In summary, these results indicate that ethanol impairs insulin signaling via blocking insulin-stimulated activation of the PI3K/Akt survival pathway and inducing apoptotic pathways in CGNs. 

## 7. Ethanol Impairs *N*-Methyl-d-Aspartate (NMDA) Signaling

NMDA, depolarizing conditions (25 mM KCl), glutamate receptor agonist, or BDNF promote survival of CGNs *in vitro* [56,92,93,94]. NMDA stimulates extracellular calcium influx through NMDA receptor and inhibits caspase-3 activation by BDNF-induced activation of tyrosine kinase and PI3-kinase pathways to exhibit anti-apoptotic effects [95,96]. The survival effects of 25 mM KCl follow BDNF-independent stimulation of MAP kinase, and activation of Cam kinase II and PI3-kinase pathways in CGNs [95]. The NMDA glutamate receptor is a multimeric, ligand-gated, transmembrane protein and constitutes four subunits: two NR1 subunits and two NR2 subunits [97]. Each NR subunit consists of four hydrophobic regions. Three (TM1, TM3, and TM4) form membrane-spanning domains while the fourth domain (TM2) makes a hairpin loop and acts as an ion channel. The TM2 domain is comprised of a calcium pore, and the TM3 domain of the NR1 subunit is associated with ethanol binding. Glycine binds at the extracellular site and the intracellular cassettes of the NR1 subunit contain sites for PKA and PKC phosphorylation. The NR1 subunit connects to the cytoskeleton through α-actinin-2, whereas the NR2 intracellular region contains phosphorylation sites for the tyrosine kinases (Fyn and Src), Cam kinase II, and PDZ-containing anchoring proteins (PSD-95, chapsyn-110/PSD-93, and SAP-102). In the physiological resting state, NMDA receptor-mediated calcium influx is blocked by magnesium ions [98], however, during membrane depolarization states, these receptors are activated by the binding of the agonist, glutamate, and co-agonists such as glycine and d-serine [99].

Ethanol alters the function of a number of neurotransmitter receptors (e.g., γ-amino butyric acid A (GABA-A), glycine, glutamate, norepinephrine, dopamine (DA), serotonin, acetylcholine, and opiate receptors), as well as transporters (adenosine, norepinephrine, DA, and serotonin transporters) [18]. Various electrophysiological and biochemical studies suggest that ethanol acts as a selective inhibitor of NMDA receptor and blocks NMDA stimulated responses in different biological systems [100,101,102,103,104,105,106]. Acute ethanol exposure blocks both NMDA receptor-mediated extracellular calcium influx and activated ionic currents in various brain regions [104,107], however, chronic ethanol exposure increases mRNA and protein levels of receptor subunits in rodent brains and cortical neurons [108,109,110,111,112,113]. *In vitro* studies with neurons in the presence of 25 μM NMDA shows that ethanol, at 20 mM concentration, inhibited NMDA responses in the hippocampus, and at 80 mM, responses were inhibited in the cerebellum and cortex [106]. In cerebellar granule neurons prepared from six to eight-day-old rat pups, ethanol treatment decreases NMDA-stimulated calcium intake and induces apoptosis in neurons [107,114].

NMDA receptor subunits have differential sensitivity to the inhibitory effects of ethanol. Follesa and Ticku [111] reported that NR2 (but not NR1) are important in altering the NMDA receptor response in chronic ethanol-treated rats [111]. Prenatal exposure to ethanol decreases both NR2A and NR2B subunit expression at prenatal day seven, and prenatal day 14 [115]. In contrast, early postnatal ethanol exposure increases NR2A subunit expression (not NR2B) at postnatal day 21 in the rat brain cortex [116]. Also studies show that recombinant heteromers containing either NR2A or NR2B subunits are more ethanol sensitive compared to heteromers containing NR2C or NR2D subunits [117,118,119]. Moreover, NMDA receptors composed of NR1/NR2B subunits are more ethanol susceptible when compared to those composed of NR1/NR2A subunits in developing cerebellar granule neurons [120]. Ethanol exposure of hippocampal slices reduces the phosphorylation of tyrosine side chains in the NR2A and/or NR2B subunits, and addition of a phosphotyrosine phosphatase inhibitor, bpV(phen), reduces the inhibitory effect of ethanol on NMDA receptor. These data suggest that the phosphorylation status of NR subunits also determines the action of ethanol [121].

## 8. Ethanol Impairs RhoA GTPase Signaling

Rho-GTPases (RhoA, Rac1 and Cdc42) are small G-proteins and act as molecular switches to transmit signals from upstream growth signals to downstream effectors [122,123]. These proteins regulate cell adhesion, differentiation, membrane trafficking, cytoskeleton rearrangement, and transcriptional activation. As positive regulators, Rac1 and Cdc42 activation (GTP-bound) promote neurite growth and formation of lamellipodia and filopodia on growth cones, whereas RhoA activation acts as a negative regulator and mediates neurite retraction and cell death. The activity of Rho-GTPases is regulated by Guanine nucleotide exchange factors (GEFs), GTPase-activating proteins (GAPs), and Guanine dissociation inhibitors (GDIs). Downstream, important effectors for active Rac1 and Cdc42 are the p21-activated kinases (PAKs), and for RhoA is Rho-kinase/ROCK. Previous studies from our laboratory and others confirm that ethanol alters the activity of Rho-GTPases and its effectors in neuronal cells [53] and non-neuronal cells [124,125,126], and regulates microtubules and F-actin cytoskeleton dynamics [127,128,129]. Using primary CGN culture from ethanol exposed seven-day-old pups, our laboratory found that moderate ethanol exposure (blood alcohol concentration 40 mM) inhibits the activation of RacI and stunts the formation of neurites, while RhoA activation at high dose of ethanol (blood alcohol 80 mM) promotes apoptosis [53]. Another study reported that ethanol inhibits BDNF-induced Rac1/Cdc42 activation and increases RhoA activation in axonal growth cones of embryonic rat hippocampal pyramidal neurons [71]. Ethanol exposure inhibited dendritic development [130] and delayed initial axon outgrowth in these cultures [131]. Recent studies with primary cultures of hippocampal neurons showed that chronic ethanol exposure decreases the levels of microtubule-associated protein-2 (MAP2), filamentous actin, and polymerized tubulin, and alters G-actin/F-actin ratio [129]. MAP2 links actin microfilaments, microtubulin and neurofilaments in dendrites and is involved in neurite initiation and microtubule stabilization [132].

Prenatal ethanol exposure during cerebral cortex development induces astroglial death [69,133], and ethanol reorganizes actin cytoskeleton and focal adhesions in astrocytes [124]. Another study carried out by Minambres *et al.* [134] indicates that ethanol activates RhoA and its effector ROCK1, promotes phosphorylation of myocin light chain (MLC) and membrane blebbing followed by cell death in astrocytes. Inhibition of both RhoA (by C3 inhibitor) and ROCK (by inhibitor Y27632) activation prevents MLC phosphorylation and the membrane blebbing induced by ethanol. These results suggest that ethanol disrupts actin cytoskeleton rearrangement and induces cell death by the activation of the RhoA/ROCK/MLC pathway in astrocytes. Similar studies further demonstrated that ethanol induces inflammation through the activation of RhoE in primary astrocytes. RhoE, acts as an antagonist of RhoA and Rac1, prevents phosphorylation of MLC, and is involved in disorganization of actin cytoskeleton. Ethanol-induced RhoE activation stimulates the IRAK/ERK/NF-κB pathway and the COX-2 expression associated with the inflammatory response. Preincubation with PKC inhibitor reduces the RhoE level and suppresses the ethanol-induced activation of IRAK, NF-κB, and the COX-2 expression suggest that PKCs are involved in stimulation of RhoE with ethanol [135]. Together, these studies suggest that a change in Rho-GTPase activity by ethanol alters the shape and mobility of cells and induces inflammation. 

## 9. Ethanol Increases Prostaglandin Production

Prostaglandins (PGs), especially prostaglandin E2 (PGE2), regulate CNS development, neural proliferation, and synaptogenesis. In prostaglandin synthesis, the enzyme phospholipase A2 (PLA2) catalyzes the formation of arachidonic acid from membrane-bound phospholipids, which is subsequently converted into prostaglandin by enzyme cyclooxygenase. *In vitro* and *in vivo* studies show that ethanol can increases endogenous PG concentrations in the CNS [136,137], and expression of COX-2 and the resulting PGE2 production in rat astrocyte cells [138]. Out of the two isoforms of COX (COX-1 and COX-2), an acute dose of ethanol (5 g/kg bwt) increases the expression COX-2 in the C4 region of the hippocampus and agranule cortex whereas chronic doses of ethanol (four days, intragastric) increases COX-2 expression in the limbic cortex, isocortex and amygdala of rat brain [139]. Although both COX forms are expressed in astrocytes and neurons, COX-2 in astrocytes is more sensitive to ethanol compared to neuronal expressed COX-2. In developing brains, astrocytes guide the migration of neurons and ethanol effects on cortical glia impair neuronal migration. In this scenario, it is suggested that some of the ethanol-induced neural damage and impairment in migration is controlled by astrocytes through the increased level of PGE2 and altered activity of COX-2 enzyme.

During cerebellar development, cGMP signaling accelerates the migration of CGNs from EGL to IGL while cAMP signaling inhibits this process. Acute ethanol administration in a mouse model of fetal alcohol spectrum disorders (FASD) increases the level of cAMP and reduces cGMP by altering phosphodiesterase 2 (PDE2) activity in neurons [140]. PDEs mediate cAMP and cGMP hydrolysis. Erythro-9-(2-hydroxy-3-nonyl) adenine (EHNA), a PDE2 specific inhibitor, reduces the inhibitory effects of ethanol on neuronal migration, whereas a broad spectrum PDE inhibitor, 3-isobutyl-1-methylanxthine (IBMX), does not reverse ethanol affected cell migration. 

Exposure of ethanol to mouse embryos, using whole embryo culture and mid-brain cultures, shows that ethanol inhibits cephalic neural tube closure, neural differentiation and proliferation, and as a result the brain is not developed properly. In these embryos, decreased levels of heat shock protein 73 (Hsp73) expression was observed in whole brains, and suggests a role for heat shock proteins in embryotoxicity due to ethanol [141].

## 10. Ethanol Targets Glycogen Synthase Kinase 3

Glycogen synthase kinase 3 (GSK3) is a multifunctional serine/therionine kinase, mainly involved in phosphorylation of glycogen synthase, an enzyme of carbohydrate metabolism. Out of the two isoforms of GSK3 (GSK3α and GSK3β), GSK3β is well studied in CNS development [16]. Subcellular localization and site-specific phosphorylation status regulate the activity of GSK3β, e.g., tyrosine phosphorylation at amino acid position 216 (tyr216) increases, while phosphorylation at serine 9 (ser9) inhibits the enzymatic activity of GSK3β. Active GSK3β associates with β-catenin, (a component of Wnt signaling) in the cytoplasm, promotes β-catenin degradation by ubiquitination, and thus prevent its nuclear translocation. In the presence of Wnt signaling stimuli, GSK3β is inactivated and β-catenin translocates to the nucleus and stimulates genes regulated by the T-cell factor (TCF)/lymphoid enhancer factor (LEF) family of transcription factors. This transcriptional effect promotes neural development, such as morphogenesis, patterning, and differentiation [142]. Under normal conditions, inactivation of GSK3β promotes the survival and proliferation of cultured cerebellar granule neural progenitor cells [143] and cortical neural progenitor cells [144]. The inhibition in GSK3β (by phophorylation at Ser9) activity is achieved by the activation of the PI3K/Akt signaling pathway stimulated by neurotrophic factors (such as insulin/IGF-I and BDNF). Since ethanol interferes with BDNF- and insulin/IGF-mediated PI3K/Akt activation in cerebellar granule neurons [70,88,145] as mentioned above, this suggests that GSK3β signaling is involved in ethanol-mediated neuronal death, migration, and differentiation [146].

Liu *et al.* [147] had previously reported that subcutaneous injection of ethanol (2.5 g/kg b.wt.) in seven-day-old C57BL/6 mouse pups significantly increases the activity of GSK3β (dephosphorylation at Ser9), activates Bax protein, and promotes apoptosis in the cerebellar cortex [147]. Similarly, another group determined that gestational ethanol exposure blocks insulin/IGF-mediated PI3-kinase/Akt activation and increases the activity of GSK3β by dephosphorylation at Ser9 in cerebellar neurons prepared from ethanol-exposed postnatal day two pups [88]. The role of GSK3β in ethanol-induced neurodegeneration is further confirmed by the administration of various GKS3β specific and non-specific inhibitors such as lithium, SB216763, alsteropaullone, and SB415286. Intraperitonial injection of lithium, an inhibitor of GSK3α and β isoforms, prior to or after ethanol-exposure effectively mitigates ethanol-induced apoptosis by inhibiting the activation of caspase-3 and -9 as well as ethanol-mediated down-regulation of p-GSK3β (Ser9), p-Akt and p-AMPK in the cerebellar cortex in seven-day-old mice pups [148,149]. In addition, one report carried out in human PNET2 cerebellar neuronal cells suggests the role of GSK3β in ethanol-mediated inhibition in neuronal cell migration [150]. This study proposed that ethanol-induced GSK3β activity phosphorylates aspartyl (asparaginyl)-β-hydroxylase (AAH) and promotes AAH degradation, which in turn inhibits cell motility. Inhibition of GSK3β activity by lithium reduces AAH degradation and reverses ethanol effects on cell migration [150]. AAH mediates neuronal motility, and is stimulated by insulin and IGF activation of PI3-kinase/Akt, or inhibition of GSK3β. Thus, posttranslational modifications (phosphorylation) and degradation of AAH protein by increased GSK3β activity promotes the inhibitory effects of ethanol on neuronal migration.

## 11. Ethanol Alters Cell Cycle Progression

Cyclin-dependent kinases (CDKs) are required for neural development and differentiation [151]. The expression of CDKs in the development of the cerebellum is tightly regulated by activators (CDK activators) and inhibitors (CDKIs) [152]. The expression level of cyclin A and CDK2 increases from postnatal day 0–6 (P0–P6), and then declines from P12–P21 in the rat cerebellum suggesting that the cyclin A/CDK2 complex is required for the proliferation of CGNs. Prenatal ethanol exposure studies demonstrate that ethanol treatment decreases the expression of cyclin A, CDK2, and up-regulates the expression of cyclin D2 and cyclin D-dependent CDKs (CDK4/6) in the cerebellum. It is suggested that down-regulation of cyclin A/CDK2 and upregulation of cyclin D/CDK4/6 in response to ethanol promotes cell cycle arrest and apoptosis in neurons, respectively [153]. As the binding of transcription factor AP-1 to the promoter region of the cyclin A gene increases its transcript expression, and prenatal ethanol exposure reduces the expression of AP-1, it can be concluded that expression of cell cycle proteins is affected by ethanol at the transcriptional level. However, in another *in vitro* study ethanol exposure of neurosphere cultures isolated from rat cerebral cortex promoted cell cycle progression. It increased the neurosphere number without inducing apoptosis. These studies indicate that ethanol promotes stem to blast cell maturation, therefore, depleting the reserve proliferation capacity of neuroepithelial cells [154]. The effects of ethanol on cell cycle progression may therefore depend on the neuronal type and stage of their development. 

## 12. Conclusion

**Figure 3:**
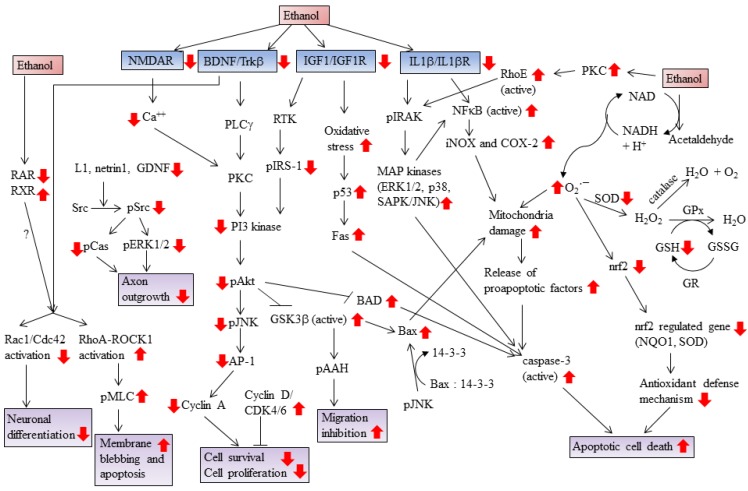
Ethanol-induced changes in signaling pathways/components (red colored arrow) in the developing cerebellum. Ethanol exposure inhibits axon outgrowth, cytoskeletal rearrangement, and neuronal differentiation via L1/Src/ERK1/2 pathways and RhoA and Rac1/Cdc42 activation. Ethanol also inhibits the cell proliferation by altering the levels of proteins required for cell cycle progression, e.g., increasing the levels of cyclin D/CDK4/6 and by decreasing the levels of cyclin A. Ethanol impairs NMDA, BDNF/TrkB, IGF/IGFR and IL1β/IL1βR-mediated pathways and their downstream signals which are required for the migration and survival of neurons. Generation of oxidative stress and reduction in the level of enzymes required for the removal of free radicals by ethanol damages mitochondria and initiates cell death in neurons.

Various animal models and *in vitro* studies suggest that pre- or postnatal ethanol exposure induces neuroanatomical, biochemical and physiological abnormalities, promotes craniofacial deformities, and behavioral dysfunction in the offspring. Ethanol induces various genes encoding cytokines and chemokines, increases free radicals, alters signaling molecule cross talk to activate apoptotic pathways, inhibits survival pathways, and furthermore, inhibit the maturation, differentiation, and migration of neurons needed for proper brain development (Figure 3).
